# Effects of state-wide implementation of the Los Angeles Motor Scale for triage of stroke patients in clinical practice

**DOI:** 10.1186/s42466-021-00128-x

**Published:** 2021-06-01

**Authors:** Stefanie Behnke, Thomas Schlechtriemen, Andreas Binder, Monika Bachhuber, Mark Becker, Benedikt Trauth, Martin Lesmeister, Elmar Spüntrup, Silke Walter, Lukas Hoor, Andreas Ragoschke-Schumm, Fatma Merzou, Luca Tarantini, Thomas Bertsch, Jürgen Guldner, Achim Magull-Seltenreich, Frank Maier, Christoph Massing, Volkmar Fischer, Michael Gawlitza, Katrin Donnevert, Hans-Michael Lamberty, Stefan Jung, Matthias Strittmatter, Silke Tonner, Johannes Schuler, Robert Liszka, Stefan Wagenpfeil, Iris Q. Grunwald, Wolfgang Reith, Klaus Fassbender

**Affiliations:** 1 Department of Neurology, Saarland University Medical Center, Kirrberger St. Bldg. 90, 66421 Homburg, Germany; 2 Zweckverband für Rettungsdienst und Feuerwehralarmierung Saar, Bexbach, Germany; 3 Department of Neurology, Klinikum Saarbrücken, Saarbrücken, Germany; 4 Department of Radiology, Klinikum Saarbrücken, Saarbrücken, Germany; 5 Institute of Clinical Chemistry, Laboratory Medicine and Transfusion Medicine, Paracelsus Medical University, Nuremberg, Germany; 6 Department of Neurology, Knappschaftsklinikum Saar, Püttlingen, Germany; 7 Department of Neurology, Caritas-Klinikum Saarbrücken St. Theresia, Saarbrücken, Germany; 8 Department of Neurology, Diakonie Klinikum Neunkirchen, Neunkirchen/Saar, Germany; 9 Department of Neurology, Knappschaftsklinikum Saar, Sulzbach, Germany; 10 Department of Neurology, DRK Krankenhaus Saarlouis, Saarlouis, Germany; 11 Department of Neurology, Marienhaus Klinikum Saarlouis-Dillingen, Dillingen, Germany; 12 Department of Neurology, SHG Klinikum Merzig, Merzig, Germany; 13 Department of Neurology, Marienhaus Klinik St. Wendel, St. Wendel, Germany; 14 Institute for Medical Biometry, Epidemiology and Medical Informatics, Saarland University, Campus Homburg, Homburg, Germany; 15 Department of Neuroscience, Medical School, Anglia Ruskin University, Chelmsford, UK; 16 Division of Imaging Science and Technology, School of Medicine, University of Dundee, Dundee, UK; 17 Department of Neuroradiology, Saarland University Medical Center, Homburg, Germany

**Keywords:** Prehospital, Large-vessel occlusion, Emergency medical services, Triage, Preclinical scale, Thrombectomy

## Abstract

**Background:**

The prehospital identification of stroke patients with large-vessel occlusion (LVO), that should be immediately transported to a thrombectomy capable centre is an unsolved problem. Our aim was to determine whether implementation of a state-wide standard operating procedure (SOP) using the Los Angeles Motor Scale (LAMS) is feasible and enables correct triage of stroke patients to hospitals offering (comprehensive stroke centres, CSCs) or not offering (primary stroke centres, PSCs) thrombectomy.

**Methods:**

Prospective study involving all patients with suspected acute stroke treated in a 4-month period in a state-wide network of all stroke-treating hospitals (eight PSCs and two CSCs). Primary endpoint was accuracy of the triage SOP in correctly transferring patients to CSCs or PSCs. Additional endpoints included the number of secondary transfers, the accuracy of the LAMS for detection of LVO, apart from stroke management metrics.

**Results:**

In 1123 patients, use of a triage SOP based on the LAMS allowed triage decisions according to LVO status with a sensitivity of 69.2% (95% confidence interval (95%-CI): 59.0–79.5%) and a specificity of 84.9% (95%-CI: 82.6–87.3%). This was more favourable than the conventional approach of transferring every patient to the nearest stroke-treating hospital, as determined by geocoding for each patient (sensitivity, 17.9% (95%-CI: 9.4–26.5%); specificity, 100% (95%-CI: 100–100%)). Secondary transfers were required for 14 of the 78 (17.9%) LVO patients. Regarding the score itself, LAMS detected LVO with a sensitivity of 67.5% (95%-CI: 57.1–78.0%) and a specificity of 83.5% (95%-CI: 81.0–86.0%).

**Conclusions:**

State-wide implementation of a triage SOP requesting use of the LAMS tool is feasible and improves triage decision-making in acute stroke regarding the most appropriate target hospital.

## Background

Stroke is a frequent cause of disability and death [1] with important medical and economic implications. When stroke is caused by large-vessel occlusion (LVO), trials provide compelling evidence that mechanical thrombectomy (MT) rather than medical treatment alone is most effective. Even so, although an estimated 10 to 25% of patients with stroke have LVO [2, 3], only a small minority (fewer than 7%) are treated with MT [2, 4, 5]. A main reason for this difference is that MT is not available at the many primary stroke centres (PSCs) but is offered only by a few specialized stroke centres (comprehensive stroke centres, CSCs).

Currently, stroke-management guidelines recommend the transport of all patients to the nearest stroke-ready hospital [6], which in most cases is a PSC not offering MT. Therefore, patients with LVO may secondarily be transferred to a CSC for thrombectomy. Importantly, compared with direct referral to a CSC, such interhospital transfers cause pronounced treatment delays ranging from 96 min to 111 min for patients with LVO [7–10]. Consistent with the “time is brain” concept [8], these delays significantly worsen clinical outcomes [9, 11]. On the other hand, not all patients with stroke should be transferred to CSCs because, apart from overwhelming already strained accident and emergency departments, bypassing PSCs could delay the administration of intravenous thrombolysis for most patients.

LVO is mostly associated with more severe stroke symptoms. Therefore, researchers have proposed the use in the field of stroke severity scales aimed at prehospitally detecting LVO; patients with LVO could then profit from direct transfer to a CSC. Recently, many LVO scales, such as the Los Angeles Motor Scale (LAMS) [12], the Rapid Arterial Occlusion Evaluation Scale (RACE) [13–15] or the A2L2 test (A, arm; L, leg) [16] have been proposed and studied by EMS in regard to their accuracy of detecting LVO.

Because the LAMS, apart from belonging to the most predictive of those scales [17, 18], requires assessment of only three motor symptoms (facial paresis, arm strength, and grip strength) [12, 19], it appears most suitable for implementation in prehospital emergency care protocols.

However, information on the effects of implementation of the LAMS for real-life triage of stroke patients is still missing. The aim of this study is to explore the effects of a state-wide triage standard operating procedure (SOP) requesting performance of the LAMS for triage decision-making in regard to the most appropriate target hospital.

## Methods

### Patients and study design

This prospective multicentre study, coordinated by the University of the Saarland, Germany, was opened on March 1, 2018, and terminated on June 30, 2018. The trial was conducted in the federal state of Saarland in Germany, a mixed urban and rural state with an area of 1004 sq. mi (2571 km^2^) and approximately 992,000 inhabitants, in the context of a state-wide network of all certified stroke centres (eight PSCs, two CSCs). All emergency calls in this state are evaluated by a single dispatch centre. Inclusion criteria were suspicion of acute stroke by the EMS personnel on-scene, age of at least 18 years, and willingness to participate. Exclusion criteria were referral modes other than via EMS and critical illness requiring immediate transfer to the nearest intensive care unit.

### Triage SOP based on the LAMS instrument

The stroke triage SOP was a rule for all EMS stations in the entire state. In this SOP, the following factors were considered in triage decision-making: (1) LAMS score (cut-off score of 4) [19]; (2) symptom onset times of 8 h or less or presence of “wake-up” stroke; and (3) quality-of-life aspects, such as severe comorbid conditions and severe prestroke dependency [20].

The triage protocol was set in operation by the central EMS coordinating authority of the state, the Zweckverband für Rettungsdienst und Feuerwehralarmierung, Saar, on May 30, 2015 (VAW MED-012) [20], and was accompanied by state-wide structured training sessions held approximately every 3 months for EMS personnel in the field and every 6 months for dispatch centre personnel in the context of the 30 educational sessions per year required for EMS personnel in the field. Moreover, the protocol was a component of the educational curriculum of the state’s EMS school.

### Endpoints

The primary endpoint was accuracy of the triage SOP in triaging stroke patients to the appropriate target hospital, PSC versus CSC. Correct triage decision was defined as a decision to transport patients with LVO to the nearest CSC and to transport patients without LVO to the nearest PSC (or to a CSC, if this was the nearest stroke-treating hospital). LVO was defined as occlusion of the intracranial internal carotid artery, the proximal (M1) segments of the middle cerebral artery, or the basilar artery.

Secondary endpoints were performance of the triage protocol with regard to either LVO or intracranial haemorrhage (ICH), the number of secondary interhospital transfers, and the sensitivity and specificity of the LAMS in detection of LVO itself. Documented stroke management metrics included times from call to (1) "on scene”, (2) hospital admission (“door”), (3) first neurologist contact, (4) start of non-contrast imaging, (5) start of vascular imaging (in case of ischaemic stroke), (6) needle, (7) door of CSC after secondary transfer, and (8) groin puncture. To determine short-term outcomes, we assessed mRS scores at discharge and mortality rates.

### Geocoding

For analysis of the potentially alternative conventional pathway of the guideline-recommended transfer to the nearest stroke-treating hospital, geocoding was performed for each patient based on postal code, as previously described [21]. This approach allowed exact identification of the level of care (PSC or CSC) offered by the nearest stroke-treating hospital to which the individual patient with stroke would normally have been transferred.

### Ethical aspects

The study protocol, the informed consent document, and the subject information document were approved by the Ethics Committee of the Medical Association of the Saarland, Germany (AZ-257/17). Informed consent was obtained from all patients or their legal representatives and was documented in the receiving hospitals. The otherwise separated datasets of the EMS and of the hospital were pseudonymized and linked only by the information from (1) the target hospital, (2) the admission date, (3) the patient’s sex, and (4) the patient’s year of birth.

### Statistical analyses

Results are reported according to Strengthening The Reporting of OBservational Studies in Epidemiology (STROBE) guidelines [22]. Group comparisons were analysed with the Mann-Whitney U Test or the Fisher exact test. Statistical analyses were performed with IBM SPSS Statistics for Windows, version 25.0.0.0 (IBM Corporation, Armonk, NY, USA).

## Results

### Demographic and medical characteristics

**Fig. 1:**
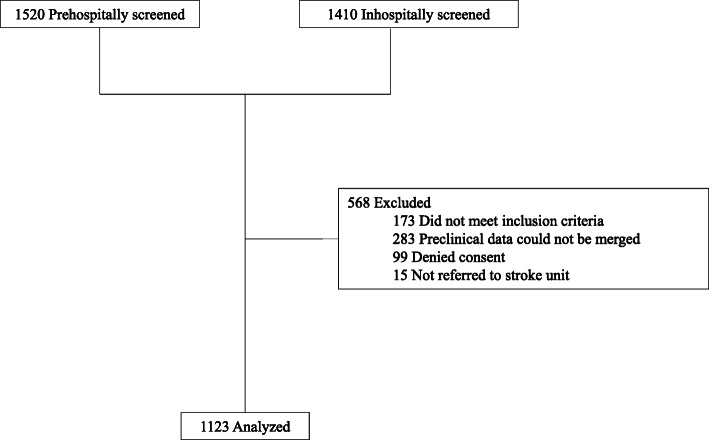
Strengthening The Reporting of Observational Studies in Epidemiology (STROBE) diagram illustrating the trial design and exclusion of patients from the study. Patients with suspected stroke were enrolled in the hospital after evaluation for inclusion and exclusion criteria and after informed consent had been obtained. For pseudonymization, separated data sets from the prehospital and in-hospital phases of acute stroke management were linked exclusively on the basis of information about the date of stroke, target hospital, sex, and the patient’s year of birth

After the 4-month study period, the study was terminated with a total of 1123 enrolled patients. Exclusion of patients is specified in Fig. 1. Baseline demographic and medical characteristics of the patients are presented in Table 1. Of the 1123 patients, 644 (57.3%) had experienced an acute ischaemic stroke, 84 patients (7.5%) had experienced a haemorrhagic stroke, and 395 patients (35.2%) had experienced stroke mimics. Of the 644 patients with ischaemic stroke, 129 (20.0%) underwent thrombolysis; and 489 underwent vascular imaging, which showed that 78 (12.1%) had LVO; 53 of these 78 patients (67.9%) were treated with MT.

**Table 1:** Demographic and medical characteristics of the study population

		Triage decision		Transport destination^**a**^	
	Total (***n*** = 1123)	CSC (***n*** = 188)	PSC (***n*** = 935)	CSC (***n*** = 424)	PSC (***n*** = 699)
Demographic and prehospital data					
Age, years; median (IQR)	78 (68–85)	74 (66–83)	79 (68–85)	76 (66–84)	80 (69–85)
Male sex, n (%)	565 (50.3)	97 (51.6)	468 (50.1)	218 (51.4)	347 (49.6)
Symptom onset to call, min; median (IQR)	55 (11–353)	19 (6–126)	68 (14–406)	36 (8–233)	74 (15–426)
LAMS, n/total n (%)					
LAMS< 4	855/1123 (80.4)	49/855 (5.7)	806/855 (94.3)	269/855 (31.5)	586/855 (68.5)
LAMS≥4	208/1123 (19.6)	135/208 (64.9)	73/208 (35.1)	140/208 (67.3)	68/208 (32.7)
NIHSS admission, median (IQR)					
All	4 (1–8)	10 (5–17)	3 (1–6)	5 (2–11)	3 (1–6)
Patients with strokes	4 (2–8)	8 (5–16)	3 (1–6)	5 (2–10)	3 (1–6)
Discharge diagnoses, n (%)					
Ischaemic stroke	644 (57.3)	119 (63.3)	525 (56.1)	254 (59.9)	390 (55.8)
*LVO stroke*	78 (6.9)	54 (28.7)	24 (2.6)	58 (13.7)	20 (2.9)
Intracranial haemorrhage	84 (7.5)	28 (14.9)	45 (4.8)	56 (13.2)	39 (5.6)
Stroke mimics	395 (35.2)	41 (21.8)	354 (37.9)	125 (29.5)	270 (38.6)
*Epileptic seizure*	85 (7.6)	21 (11.2)	64 (6.8)	38 (9.0)	47 (6.7)
*Migraine*	11 (1.0)	1 (0.5)	11 (1.2)	3 (0.7)	8 (1.1)
*Vestibulopathy*	31 (2.8)	0	31 (3.3)	9 (2.1)	22 (3.1)
*Infection*	52 (4.6)	4 (2.1)	48 (5.1)	19 (4. 5)	33 (4.7)
*Exsiccosis*	17 (1.5)	0	17 (1.8)	4 (0.9)	13 (1.9)
*Delirium*	31 (2.8)	0	31 (3.3)	8 (1.9)	23 (3.3)
*Hypertensive crisis*	6 (0.5)	1 (0.5)	5 (0.5)	2 (0.5)	4 (0.6)
*Peripheral facial palsy*	11 (1.0)	0	11 (1.2)	6 (1.4)	5 (0.7)
*Intoxication*	6 (0.5)	1 (0.5)	5 (0.5)	2 (0.5)	4 (0.6)
*Syncope*	18 (1.6)	1 (0.5)	17 (1.8)	4 (0.9)	14 (2.0)
*Other*	86 (7.7)	12 (6.4)	74 (7.9)	31 (7.3)	55 (7.9)

*CSC* Comprehensive stroke centre, *PSC* Primary stroke centre, *IQR* Interquartile range, *LAMS* Los Angeles Motor Scale, *NIHSS* National Institutes of Health Stroke Scale, *mRS* Modified Rankin Scale, *LVO* Large-vessel occlusion

^a^ Including CSCs serving as PSCs if they are the closest stroke centre at all

### Feasibility and performance of the LAMS-based triage SOP in triage of stroke patients

This study demonstrated the feasibility of state-wide implementation of a LAMS-based triage SOP with high adherence. Only 60 of 1123 patients (5.3%) had no documented LAMS score, and 941 of the 1123 patients (83.8%) were triaged according to the LAMS score (Table 1).

The triage SOP allowed triage to the appropriate hospital with a sensitivity of 69.2% (95% confidence interval (95%-CI): 59.0–79.5%) and a specificity of 84.9% (95%-CI: 82.6–87.3%) (Table 2). In contrast, if the current guidelines had been adhered to (transfer to the nearest stroke-treating hospital), patients with LVO would have been correctly triaged with a sensitivity of 17.9% (95%-CI: 9.4–26.5%) and a specificity of 100% (95%-CI: 100–100%).

**Table 2:** Performance of the triage SOP and modelled conventional care assessed via geocoding in patients with LVO and with LVO or ICH^a^

	Triage SOP (***n*** = 968)	Modelled conventional care^**b**^ (***n*** = 968)
LVO, n/total n (%)		
Sensitivity	54/78 (69.2; 59.0–79.5)	14/78 (17.9; 9.4–26.5)
Specificity	756/890 (84.9; 82.6–87.3)	890/890 (100; 100–100)
Positive Predictive Value	54/188 (28.7; 22.3–35.2)	14/14 (100; 100–100)
Negative Predictive Value	756/780 (96.9; 95.7–98.1)	890/954 (93.3; 91.7–94.9)
LVO or ICH, n/total n (%)		
Sensitivity	82/162 (50.6; 42.9–58.3)	38/162 (23.5; 16.9–30.0)
Specificity	700/806 (86.8; 84.5–89.2)	806/806 (100; 100–100)
Positive Predictive Value	82/188 (43.6; 36.5–50.7)	38/38 (100; 100–100)
Negative Predictive Value	700/780 (89.7; 87.6–91.9)	806/930 (86.7; 84.5–88.9)

*SOP* Standard operating procedure, *LVO* Large-vessel occlusion, *ICH* Intracranial haemorrhage

^a^ 60 of 1123 (5.3%) patients had no documented LAMS score, and 155 of 644 (24.1%) ischaemic stroke patients did not undergo vascular imaging; ^b^ Conventional care was the guideline-recommended transfer to the nearest stroke centre, as calculated via geocoding in each patient. CSCs served as PSCs if they were the nearest stroke centre

95% confidence intervals are reported in brackets

### Performance of the LAMS in identifying LVO

**Fig. 2:**
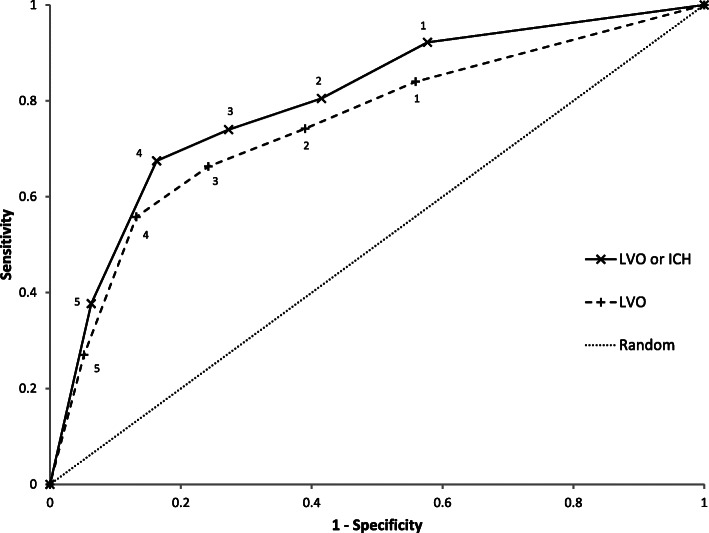
Receiver operating characteristic curves of the Los Angeles Motor Scale at various cut-off scores for diagnosing large-vessel occlusion (LVO) selectively (dashed line) with an area under the curve of 0.798, or for diagnosing LVO or intracerebral haemorrhage (solid line) with an area under the curve of 0.751. Diagonal segments are produced by ties. The dotted line depicts randomness

The LAMS itself, at a cut-off value of 4, exhibited a sensitivity of 67.5% (95%-CI: 57.1–78.0%) and a specificity of 83.5% (95%-CI: 81.0–86.0%) in detecting LVO (Table 3). Moreover, this scale exhibited a sensitivity of 56.6% (95%-CI: 48.9–64.3%) and a specificity of 86.7% (95%-CI: 84.3–89.1%) in detecting either LVO or ICH (Table 3). The receiver operating characteristic (ROC) curves obtained with a wider range of LAMS cut-off scores are displayed in Fig. 2 and suggest that the cut-off value of 4 is appropriate.

**Table 3:** Performance of the LAMS tool (cut-off value, ≥4) in identification of LVO and LVO or ICH

	All patients^**a**^ (***n*** = 920)		Patients with stroke^**b**^ (***n*** = 547)	
Variable, n/total n (%)	LVO	LVO or ICH	LVO	LVO or ICH
Sensitivity	52/77 (67.5; 57.1–78.0)	90/159 (56.6; 48.9–64.3)	52/77 (67.5; 57.1–78.0)	90/158 (57.0; 49.2–64.7)
Specificity	704/843 (83.5; 81.0–86.0)	660/761 (86.7; 84.3–89.1)	366/470 (77.9; 74.1–81.6)	323/389 (83.0; 79.3–86.8)
Positive predictive value	52/191 (27.2; 20.9–33.5)	90/191 (47.1; 40.0–54.2)	52/156 (33.3; 25.9–40.7)	90/156 (57.7; 49.9–65.4)
Negative predictive value	704/729 (96.6; 95.3–97.9)	660/729 (90.5; 88.4–92.7)	366/391 (93.6; 91.2–96.0)	323/391 (82.6; 78.9–86.4)

*LAMS* Los Angeles Motor Scale, *LVO* Large-vessel occlusion, *ICH* Intracranial haemorrhage; Independently of having received vascular imaging, patients with ICH or stroke mimics are classified as LVO-negative

^a^ 60 of 1123 (5.3%) patients had no documented LAMS score, and 155 of 644 (24.1%) ischaemic stroke patients did not undergo vascular imaging; ^b^ 38 of 728 (5.2%) patients had no documented LAMS score, and 155 of 644 (24.1%) ischaemic stroke patients did not undergo vascular imaging

95% confidence intervals are reported in brackets

Similar results were obtained when the evaluation included ICH as a condition for triage to a CSC (Table 3). In accordance with this high triage accuracy, only 14 of the 78 patients with LVO (17.9%) required secondary transfer.

### Stroke management metrics and short-term outcomes

Stroke management metrics are displayed in Table 4. The median call-to-needle times for patients transferred to a PSC were shorter than those for patients transferred to a CSC, and, conversely, call-to-groin puncture times were longer for patients transferred to a PSC than for those transferred to a CSC (Table 4).

**Table 4:** Stroke management metrics

	Total (***n*** = 1077)	CSC (***n*** = 400)	PSC (***n*** = 677)	***p***-value
Stroke-management metrics, min; median (IQR)				
Time from call to				
On-scene	10 (8–13)	9 (7–12)	10 (8–13)	0.105
Door^a^	49 (41–59)	51 (41–61)	48 (40–59)	0.008
First contact to neurologist	51 (42–62)	51 (42–62)	52 (43–62)	0.739
Non-contrast imaging	75 (60–99)	73 (58–104)	76 (62–96)	0.698
Initial vascular imaging	75 (62–99)	74 (60–99)	76 (64–98)	0.005
Needle	87 (67–100)	93 (73–103)	79 (66–95)	0.017
Groin puncture	112 (95–135)	105 (88–121)	292 (230–515)	< 0.001
Time from door to				
Non-contrast imaging	23 (13–42)	18 (11–43)	26 (16–40)	< 0.001
Initial vascular imaging	21 (15–38)	19 (14–33)	27 (20–42)	< 0.001
Needle	35 (27–49)	40 (29–50)	32 (26–48)	0.054
Groin puncture	57 (48–85)	53 (47–74)	233 (172–446)	< 0.001

*CSC* Comprehensive stroke centre, *PSC* Primary stroke centre, *IQR* Interquartile range

^a^ Time to admission at the first receiving hospital

In agreement with their lower LAMS scores, patients admitted to PSCs had better discharge modified Rankin scale (mRS) scores (1; interquartile range (IQR), 0–3) than did those admitted to a CSC (mRS score, 2; IQR, 1–5; *p* < 0.001). In addition, mortality rates were lower for patients transferred to a PSC (22, 4.8%) than for those transferred to a CSC (41, 11.9%; *p* < 0.05). Similarly, among patients with a stroke diagnosis, those admitted to PSCs had better discharge mRS scores (1; IQR, 0–3) than did those admitted to a CSC (mRS score, 2; IQR, 1–5; *p* < 0.001); they also exhibited lower mortality rates (12 patients, 3.6%) than did those admitted to a CSC (22 patients, 8.4%; *p* < 0.05).

## Discussion

The current guideline-recommended practice of transferring each patient to the nearest stroke-treating hospital, usually a hospital not offering thrombectomy, may delay or even preclude thrombectomy for patients with LVO. The results show that state-wide implementation of an SOP requesting the use of LAMS for triage decisions is feasible with high adherence and that this intervention can improve triage decision-making in regard to the appropriate target hospital in clinical practice.

Our evaluation of the effects of the state-wide implementation of an EMS SOP based on the LAMS achieved a sensitivity of 69.2% and a specificity of 84.9% in transferring patients with LVO to the appropriate target hospital. In accordance with such a high accuracy of triage decisions, we observed a low rate of secondary transfers (only 17.9% of LVO patients). In contrast, the conventional, guideline-recommended approach of transferring every patient to the nearest stroke-treating hospital, as determined by geocoding for each of the patients, would have achieved a sensitivity of only 17.9% and a specificity of 100%, a finding supporting the benefit of using the LAMS-based SOP. (The high specificity achieved by the modelled conventional pathway can be explained by the fact that “non-LVO” patients would always arrive at the “correct” hospital: the nearest one, regardless of the MT options available there.) Thus, this finding strongly suggests that implementation of a protocol using the LAMS for triage decision-making is superior to the conventional approach of transporting every patient to the nearest hospital.

Similar values were obtained when ICH was included as a “target condition” for triage to a CSC. Although increasing evidence indicates that the “time-is-brain” concept is also valid for patients with haemorrhagic stroke and that these patients could also benefit from rapid specialist treatment at a CSC [12, 23], evidence from randomized studies regarding primary transfer to CSCs is still scarce [24].

Regarding the LAMS itself, this instrument detected LVO with a sensitivity of 67.5% and a specificity of 83.5% and detected either LVO or ICH with a sensitivity of 56.6% and a specificity of 86.7%. Calculating the accuracy of a variety of alternative LAMS cut-off scores confirmed that the cut-off score of 4 is appropriate. Thus, these are within the range of results of most earlier validation studies on the LAMS. E.g., a subgroup of 94 patients from the FAST-MAG trial who underwent vascular imaging found that the LAMS detected LVO with a sensitivity of 76% and a specificity of 65% [12], and two European studies reporting sensitivities of 63 and 38%, and specificities of 84 and 93%, respectively [17, 18].

However, these earlier studies did not use their LAMS results for triaging patients in clinical practice. At the same time, the results also corroborate the conclusions of previous meta-analyses indicating that all of the LVO scales tested still miss a substantial proportion of LVOs [25, 26].

While the strengths of this study are the pseudonymized linkage of separated prehospital and in-hospital data sets, and the high degree of data completeness, one limitation of the study is the lack of a control group. The alternative of retrieving historical data before implementing the SOP may, however, have caused a bias by secular effects, and randomization was not possible in this state-wide EMS rule. However, geocoding allowed us to exactly identify the level of care offered by the nearest stroke-treating hospital in each patient if the conventional practice had been applied. Furthermore, in this real-life-study, vascular imaging has not been performed in all patients admitted to the ten hospitals, thus resulting in omission of 24% of stroke patients from evaluation. In the future, triage accuracy may be further enhanced by improved scales, by the inclusion of additional factors such as screening tools for treatment eligibility or for mimicking conditions [27], or by additional telemedical consultation with the CSC team [16].

## Conclusion

The prehospital identification of stroke patients with LVO that should be directly transported to a thrombectomy capable centre is an unsolved medical problem. Here, we show for the, to our knowledge, first time that a state-wide EMS SOP for stroke management requesting determination of the LAMS score is feasible with high adherence and can indeed be beneficial in triage decision-making regarding the most appropriate target hospital.

## Data Availability

The datasets used and analysed during the current study are available from the corresponding author upon reasonable request.

## Abbreviations

95%-CI: 95% confidence interval
CSC: Comprehensive stroke centre
EMS: Emergency medical service
ICH: Intracranial haemorrhage
IQR: Interquartile range
LAMS: Los Angeles Motor Scale
LVO: Large-vessel occlusion
mRS: Modified Rankin Scale for Neurologic Disability
MT: Mechanical thrombectomy
NIHSS: National Institutes of Health Stroke Scale
PSC: Primary stroke centre
ROC: Receiver operating characteristic
SOP: Standard operating procedure
